# Ethylene Response Factor TERF1, Regulated by ETHYLENE-INSENSITIVE3-like Factors, Functions in Reactive Oxygen Species (ROS) Scavenging in Tobacco (*Nicotiana tabacum* L.)

**DOI:** 10.1038/srep29948

**Published:** 2016-07-20

**Authors:** Hongbo Zhang, Ang Li, Zhijin Zhang, Zejun Huang, Pingli Lu, Dingyu Zhang, Xinmin Liu, Zhong-Feng Zhang, Rongfeng Huang

**Affiliations:** 1 Tobacco Research Institute, Chinese Academy of Agricultural Sciences, Qingdao 266101, China; 2 Biotechnology Research Institute, Chinese Academy of Agricultural Sciences, Beijing 100081, China; 3 Institute of Crop Sciences, Chinese Academy of Agricultural Sciences, Beijing 100081, China; 4 The Institute of Vegetables and Flowers, Chinese Academy of Agricultural Sciences, Beijing 100081, China; 5 Institute of Plant Biology, School of Life Sciences, Fudan University, Shanghai 200433, China

## Abstract

The phytohormone ethylene plays a crucial role in the production and accumulation of reactive oxygen species (ROS) in plants under stress conditions. Ethylene response factors (ERFs) are important ethylene-signaling regulators functioning in plant defense responses against biotic and abiotic stresses. However, the roles of ERFs during plant adapting to ROS stress have not yet been well documented. Our studies previously reported that a tomato ERF transcription factor TERF1 functions in the regulation of plant ethylene responses and stress tolerance. Here, we report our findings regarding the roles of TERF1 in ROS scavenging. In this study, we revealed that the transcription of *TERF1* is regulated by upstream EIN3-like (EIN3, ethylene-insensitive 3) regulators LeEIL3 and LeEIL4 in tomato (*Solanum lycopersicum*), and is also inducible by exogenous applied ROS-generating reagents. Ectopic expression of *TERF1* in tobacco promoted the expression of genes involved in oxidative stress responses, including carbonic anhydrase functioning in hypersensitive defense, catalase and glutathione peroxidase catalyzing oxidative reactions, and GDP-D-mannose pyrophosphorylase functioning in ascorbic acid biosynthesis, reduced the ROS content induced by ethylene treatment, and enhanced stress tolerance of tobacco seedlings to hydrogen peroxide (H_2_O_2_). Cumulatively, these findings suggest that TERF1 is an ethylene inducible factor regulating ROS scavenging during stress responses.

ROS production in plants occurs during normal cell metabolism or response to stress conditions through activation of NADPH oxidases^1^,^2^,^3^,^4^,^5^,^6^,^7^. Virtually all biotic and abiotic stresses could induce a rapid ROS increase in plants, which provokes the oxidation of cellular components to trigger specific plant responses, such as stomatal closure and programmed cell death^8^,^9^,^10^,^11^. To maintain the ROS levels under control, plants have developed various protective mechanisms for ROS scavenging^8^,^11^,^12^. On the other hand, ROS could act as signaling molecules to transmit physiological signals to the nucleus by oxidizing upstream components of the signaling pathway to ultimately tune up the tolerance responses against stresses^7^,^11^,^13^,^14^.

The gaseous phytohormone ethylene, which regulates plant growth, development and stress responses, is associated with ROS production under stress conditions^15^,^16^,^17^,^18^,^19^,^20^,^21^,^22^. The ethylene-overproducing mutants *eto1* and *eto3* are more sensitive to ROS stress and exhibit an enhanced ROS-induced spread of cell death^23^, while the ethylene-insensitive mutants *etr1* and *ein3* are highly tolerant to ROS stress^24^,^25^. Accordingly, ethylene synthesis was shown to be required for “ROS burst” induced cell death^24^,^25^,^26^,^27^. These facts indicate that ethylene is a positive regulator of ROS production and ROS-induced spread of cell death. On the other hand, the ethylene-induced ROS production in plants was mediated by the NADPH oxidases, which are regulated by multiple stress factors^28^,^29^. And, stress-induced ROS production could rapidly promote ethylene production^30^,^31^,^32^,^33^. These evidences implied a reciprocal regulation between ethylene and ROS production.

The ethylene-signaling pathway has been well studied^21^,^34^. Gaseous ethylene is perceived by a family of receptors that negatively regulates ethylene signaling^22^,^34^,^35^,^36^,^37^. Then, the negative regulator CTR1 transmits the signal onto the integral membrane protein EIN2 through modulating a cascade of mitogen-activated protein kinases (MAPKs) to activate the transcription factor EIN3, the direct regulator of ethylene response factors (ERFs) that bind the GCC-box in the promoters of ethylene inducible defense-related genes^20^,^35^,^38^. A number of components of ethylene-signaling pathway have been found to be involved in the ROS responses^28^,^33^,^39^,^40^. The ethylene receptor ETR1 was shown to be a critical node in mediating the cross-talk between ethylene and ROS signaling in stomatal guard cells^28^. The MAPK cascade is also suggested to be shared by both ethylene and ROS signaling^32^,^41^,^42^. And, mutation in ethylene receptor ETR1 and transcription factor EIN3 could greatly increase the ROS tolerance of Arabidopsis^13^,^25^,^28^,^33^. The plant specific ERF transcription factors are crucial regulators in plant responses to both biotic and abiotic stresses and regulate a number of stress responsive genes through binding the cis-elements in their promoters, including GCC-box, DRE/CRT, CE1, JERE and CT-rich *etc*.^38^,^43^,^44^,^45^,^46^,^47^. Recent studies also showed that ERFs are correlated with ROS production^39^,^48^,^49^,^50^. For instance, Arabidopsis AtERF15 and AtERF71, pepper CaPF1, and tomato JERF3, which function in plant biotic and/or abiotic stress responses, were all reported to be involved in the regulation of ROS production or accumulation^39^,^48^,^49^,^51^. However, the regulatory relationship between ERFs and ROS production during stress responses is still unclear.

Our studies previously revealed that the tomato ERF transcription factor TERF1 is inducible by ethylene, salinity, drought, cold, and ABA, and that TERF1 functions in plant ethylene responses and diverse stress responses^52^,^53^,^54^,^55^. In this study, we analyzed the transcriptional regulation of *TERF1* by ethylene signaling regulators and ROS-generating reagents, and investigated the roles of TERF1 in regulating plant ROS tolerances, which have provided valuable information to unravel the underlying mechanism by which ERF transcription factors coordinating plant stress tolerance and ROS responses.

## Results

### Regulation of *TERF1* transcription by LeEILs

**Figure 1:**
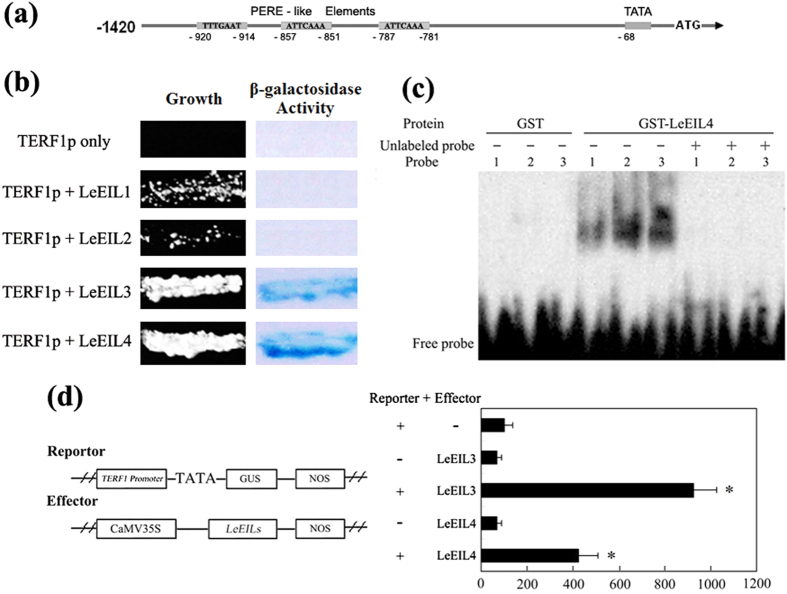
Molecular interaction between LeEILs and *TERF1* promoter. (**a**) Schematic diagram of *TERF1* promoter, showing the position of PERE-like elements. TATA, TATA-box; ATG, initiation codon. (**b**) Binding of LeEILs to the PERE-like-element-containing fragment from *TERF1* promoter in yeast. TERF1p, PERE-like-element-containing fragment. Transformants could grow on the selective medium (Growth, lacking histidine but containing 30 mM 3-AT) were subjected to filter-lifted β-galactosidase activity (β-galactosidase Activity). (**c**) *In vitro* interaction between LeEIL4 and the PERE-like elements in *TERF1* promoter in gel-shift assay. GST-LeEIL4 indicates GST-tagged LeEIL4 protein, GST is control. Number 1, 2 and 3 indicate probes containing the PERE-like element at position -920–920, -857–851 and -787–781, respectively, and the corresponding unlabeled probes (Unlabeled probe) were used as competitive probes. Free probe indicates unbound probes. (**d**) Binding of LeEIL3/4 to *TERF1* promoter in tobacco leaves. Left panel shows the structures of reporter and effector vectors, the effector vectors are driven by 35S promoter (CaMV35S). NOS, NOS terminator. The relative GUS activity values are the average of three independent triplicates. The GUS activity of non-effector control was set as 100. Error bars indicate ±SD. Asterisks indicate significant differences from the control (*P* < 0.005, Student’s *t* test).

Previous studies on the promoters of ethylene responsive genes identified an element of PERE (primary-ethylene-response element) that is specifically recognized by the ethylene signaling component EIN3 (ETHYLENE-INSENSITIVE3) or EIL1/2 (EIN3-like1/2)^35^,^56^. As shown in Fig. 1a, the promoter of *TERF1* was found to possess three PERE-like elements (ATTCAAA, - 781 to -787; ATTCAAA, -851 to -857; TTTGAAT, -914 to -920) besides other stress responsive elements^53^. The PERE-like fragment of *TERF1* promoter shows high similarity to those identified in other ethylene responsive genes, such as *Arabidopsis* PERE element (GGATTCAAG)^56^ and the PERE element (TTCAAAT) in *Os-EBP89* promoter^57^,^58^. This evidence indicates that the transcription of *TERF1* should be regulated by EIN3 or EIL1/2 through these PERE-like elements. To investigate this hypothesis, *Agrobacterium*-mediated transient *in vivo* transcription activation assay and yeast-one-hybrid were employed.

In tomato, four homologues of Arabidopsis EIN3 have been identified. They are LeEIL1, LeEIL2, LeEIL3 and LeEIL4^59^,^60^, which share a similarity about 70% to Arabidopsis EIN3 in amino acid sequence (Fig. S1). In the yeast-one-hybrid assay, LeEIL3 and LeEIL4 were able to activate the expression of histidine and *lacZ* reporter genes controlled by the PERE-like-element-containing fragment from *TERF1* promoter, and allowed the yeast to grow on selective medium lacking histidine, but containing 30 mM 3-AT (3-amino-1,2,4-triazole, a competitive inhibitor of histidine) and to display high β-galactosidase activity, while LeEIL1 and LeEIL2 exhibited a poor capability in growth on the selective medium or in the activation of β-galactosidase activity (Fig. 1b). Further gel-shift assay indicated an *in vitro* interaction between LeEIL4 and the PERE-like elements in *TERF1* promoter (Fig. 1c). In the *Agrobacterium*-mediated transient *in vivo* transcription activation assay with tobacco leaves, the effector vectors expressing *LeEIL3* and *LeEIL4* could strongly activate the expression of *TERF1*-promoter-controlled *GUS* gene, which resulted in an increase in the GUS activity as high as 9 times of that by the control vector (Fig. 1d). These data suggested that the transcription of *TERF1* is under the regulation of LeEIL3 and LeEIL4.

### Transcription of *TERF1* is ROS inducible

**Figure 2:**
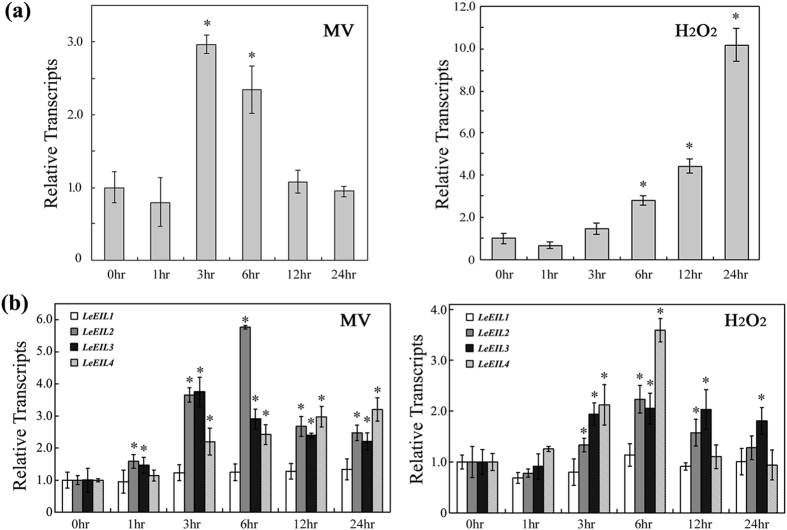
Induction of *TERF1* (**a**) and *LeEILs* (**b**) expression by oxidative reagents MV and H_2_O_2_. The transcript values are the average of three independent triplicates. The transcription level of each gene in untreated samples (0 hr) was set as “1”. Actin gene was used as internal control. Error bars indicate ±SD. Asterisks indicate significant differences from the data of untreated sample (*P* < 0.005, Student’s *t* test).

We previously demonstrated that transcription of *TERF1* was induced by multiple environmental factors, and that ectopic expression of *TERF1* could increase plant tolerance to abiotic stresses^52^,^53^,^54^,^55^. Abiotic stress responses are highly correlated with ROS production^9^,^10^,^17^,^61^, and this fact promoted us to determine the involvement of TERF1 in ROS responses. We first analyzed the transcription response of *TERF1* in response to methyl viologen (MV, ROS-generating reagent) in tomato leaves, and the results showed that MV treatment caused an increase in *TERF1* transcription by 3 folds after 3–6 hr of treatment (Fig. 2a). Further studies revealed that hydrogen peroxide (H_2_O_2_) could also induce the transcription of *TERF1*, which showed a steady increase during the H_2_O_2_ treatment and could be induced by over 10 fold after 24 hr of treatment (Fig. 2a). These data showed that *TERF1* is responsive to ROS induction. Interestingly, our study suggested that the transcription of *LeEILs* is also responsible to treatments with methyl viologen or H_2_O_2_ (Fig. 2b), supporting an involvement in the ROS stress responses.

### Expression of *TERF1* enhanced H_2_O_2_ tolerance of tobacco seedling

**Figure 3:**
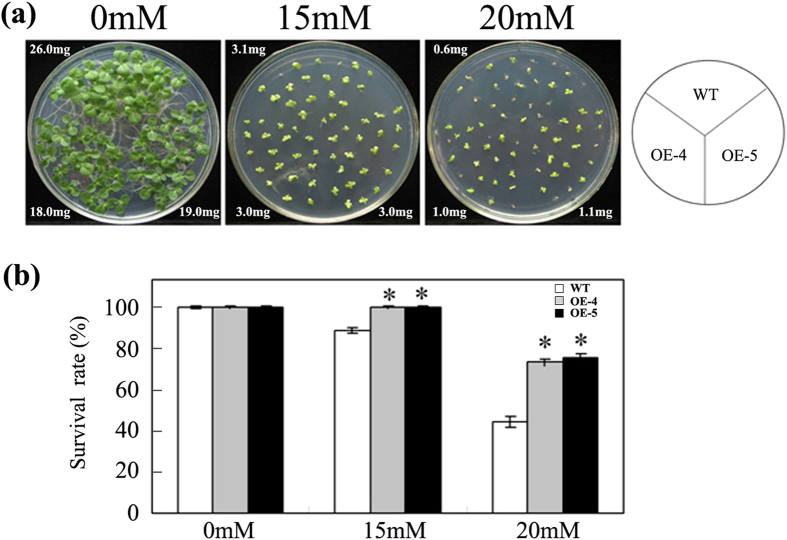
Expression of *TERF1* enhances tobacco tolerance to H_2_O_2_ during seedling development. **(a)** Seedlings cultured on MS medium (0.6% agar) with indicated concentration of H_2_O_2_ after germination. The average fresh weight per seedling (mg) was indicated at the corners of each plant set. **(b)** Survival rate of tobacco seedlings surveyed after culture on H_2_O_2_-containing MS medium for 14 days. Each value represents the average of at least 50 plants of three replicates. WT, wild type tobacco; OE-4/5, *TERF1*-expressing tobacco lines. Error bars indicate ±SD. Asterisks indicate significant differences from the data of wild type plants (*P* < 0.005, Student’s *t* test).

To determine the roles of TERF1 in ROS responses, the *TERF1*-expressing tobacco plants, which were developed previously^52^,^53^, were applied to study the effects of ROS treatment on seedling development, and H_2_O_2_ was used as ROS treatment reagent. After 10 days of culture on medium with 15 mM H_2_O_2_, the development of both wild type and transgenic tobacco seedlings were arrested, resulting in smaller and yellow leaves, but no significant difference between them could be observed except that a few wild type seedlings start dying. After 10 days of culture on medium with 20 mM H_2_O_2_, only a few wild type seedling were alive and the rest were died or dying. In the representative plates shown in Fig. 3a, only 4 wild type seedlings were alive while most seedlings of the transgenic lines were alive except for 3 of them. A greater reduction in the average seedling weight was also observed in wild type plants under the treatment with 20 mM H_2_O_2_ (Fig. 3a). Statistic data showed that the survival rate of wild type seedlings was less than 50% upon treatment with 20 mM H_2_O_2_ for 14 d, while that of the transgenic tobacco seedling could reach ~80% (Fig. 3b). This indicated that overexpression of *TERF1* could significantly increase the tolerance of tobacco seedlings to H_2_O_2_.

### Overexpression of *TERF1* decreases ethylene-induced ROS accumulation in tobacco leaves

**Figure 4:**
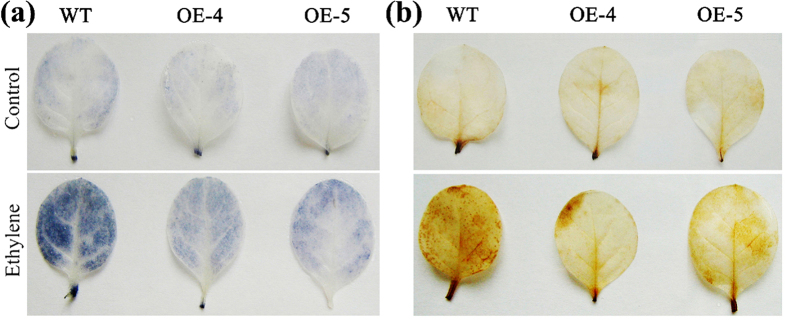
Roles of TERF1 in ROS scavenging. (**a**) NBT staining for superoxide in tobacco leaves. (**b**) DAB staining for H_2_O_2_ in tobacco leaves. Control indicates untreated seedlings. Ethylene indicate ethylene-treated seedling. Approximately 15 seedlings were used for each line, and the representative pictures of three replicates are shown.

Our previous and present studies have shown that *TERF1* is inducible by multiple environmental factors including ROS and ethylene^52^,^54^, indicating that TERF1 may be involved in the regulation of ROS production by ethylene. Therefore, the roles of TERF1 in regulating ethylene-induced ROS responses were investigated with the *TERF1*-expressing tobacco plants^52^,^53^. Plant produced ROS includes H_2_O_2_, singlet oxygen, superoxide, and the hydroxyl radical^12^,^62^, and the contents of ethylene-induced superoxide and H_2_O_2_ in *TERF1*-expressing tobacco seedlings were examined by chemical staining with nitroblue tetrazolium (NBT) and diaminobenzidine tetrahydrochloride (DAB) respectively. As shown in Fig. 4a, NBT staining of superoxide showed that superoxide accumulation kept at low levels under normal conditions and no obvious difference could be observed between wild type and transgenic seedlings. After exposure to ethylene gas (200 ppm) for 3 hr, the superoxide accumulation increased to a much higher level in wild type plants, but remained at a much lower level in the *TERF1*-expressing seedlings. Similarly, DAB staining of H_2_O_2_ showed low H_2_O_2_ levels in both wild type and *TERF1*-expressing plants under normal growth condition. And, the H_2_O_2_ content accentuated to a much higher level in wild type plants upon ethylene treatment, but remained at a much lower level in the *TERF1*-expressing plants (Fig. 4b). These results suggested that TERF1 could decrease ethylene-induced ROS accumulation.

### Expression of *TERF1* activated the expression of oxidative-related genes in tobacco

**Figure 5:**
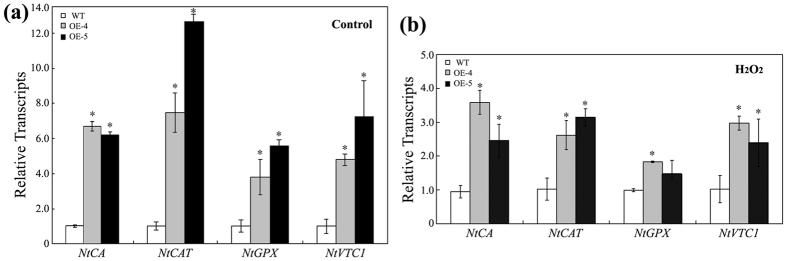
Expression of *TERF1* activates the expression of ROS response related genes in tobacco under normal condition (**a**) and H_2_O_2_ treatment (**b**). The transcript value for each gene is the average of three independent replicates. *NtCA*, carbonic anhydrase gene; *NtCAT*, catalase gene; *NtGPX*, glutathione peroxidase gene; *NtVTC1*, GDP-D-mannose pyrophosphorylase gene. The transcription level of each gene in wild type tobacco was set as “1”. *NtActin* was used as internal control. WT, wild type tobacco; OE-4/5, *TERF1*-expressing tobacco lines. Error bars indicate ±SD. Asterisks indicate significant differences from the data of wild type plants (*P* < 0.005, Student’s *t* test).

In order to reveal the mechanism by which TERF1 regulates plant responses to ethylene-induced ROS production, we analyzed the roles of TERF1 in regulating oxidative-related genes, including *NtCA* encoding a carbonic anhydrase functioning in hypersensitive defense^63^, *NtCAT* and *NtGPX* encoding catalase and glutathione peroxidase respectively that use H_2_O_2_ as an electron acceptor to catalyze oxidative reactions^64^,^65^, and GDP-D-mannose pyrophosphorylase gene (referred to as NtVTC1) functioning in ascorbic acid biosynthesis^66^,^67^. The qRT-PCR assays showed that the expression of *NtCA* and *NtCAT* was constitutively increased approximately 6 folds in the *TERF1*-expressing plants compared to that in wild type plants (Fig. 5a). And, the expression levels of *NtGPX* and *NtVTC1* were also increased by over 4 folds in the *TERF1*-expressing tobacco plants (Fig. 5a). The transcripts of these oxidative-related genes remained higher levels in the *TERF1*-expressing tobacco plants even after treatment with H_2_O_2_ (Fig. 5b). These data support the hypothesis that TERF1 is involved in the regulation of oxidative-related genes, and are helpful to explain the roles of TERF1 in regulating plant responses to ethylene-induced ROS production.

## Discussion

ROS production is involved in plant stress responses to both biotic and abiotic stresses, and is modulated by signaling pathways of multiple phytohormones, including ABA, salicylic acid (SA) and ethylene *etc.*^6^,^16^,^68^,^69^,^70^,^71^. Ethylene signaling pathway plays important roles in the regulation of ROS production^24^,^25^,^26^,^27^,^33^, and a number of the downstream ERF transcription factors in ethylene pathway function as regulators coordinating both biotic and abiotic stress responses^39^,^48^,^49^,^50^,^51^. On the other hand, stress responses in plants are highly related to the production and accumulation of ROS, which involves the regulation by ethylene signaling pathway^15^,^16^,^26^,^27^,^68^. Our previous studies showed that an ethylene-inducible tomato ERF transcription factor TERF1 modulates plant tolerance to salt, drought, and cold^52^,^53^,^54^,^55^. In this study, we further demonstrate that TERF1 is regulated by EILs and plays a role in ROS scavenging in plants, which has extended the understanding to ERF transcription factor in regulating plant stress responses.

Ethylene signal is perceived by a family of membrane receptors, and transducted by the downstream components CTR1, EIN2, EIN3 and ERF transcription factors^20^,^22^,^26^,^34^,^35^,^36^,^38^. EIN3 as well as its homologues EILs could directly bind the promoters of ERF genes through the PERE elements to regulate their expression^35^,^56^,^58^. In this study, we analyzed the capability of tomato EILs (LeEIL1, LeEIL2, LeEIL3, and LeEIL4) in binding *TERF1* promoter and regulating its expression. Yeast-one-hybrid and gel-shift assays indicates that LeEIL3 and LeEIL4 could bind *TERF1* promoter through the PERE-like elements. And, the *Agrobacterium*-mediated transient activation assay also revealed that LeEIL3 and LeEIL4 could activate *TERF1*-promoter controlled *GUS* expression *in vivo*. These findings showed that TERF1 might function as a factor regulating a subset of ethylene signaling pathway downstream of LeEIL3 and LeEIL4. On the other hand, the transcription of *TERF1* as well as *LeEILs* could also be induced by ROS-generating reagents, which indicates their involvement in plant ROS responses. TERF1 was previously evidenced to be an ethylene-inducible factor that acts as a regulator in various plant abiotic stress tolerance^52^,^53^,^54^,^55^. Whereas, ROS production is always provoked under abiotic stresses, in which ethylene pathway plays important regulatory roles^17^,^25^,^33^,^39^. These evidences suggest that TERF1 may function as coordinative regulator in responses to abiotic stress- and ethylene-induced ROS stress. Investigating the roles of TERF1 in regulating ROS responses is much helpful to understand the mechanism underlying plant stress responses.

By analyzing the ROS accumulation in *TERF1*-expressing tobacco plants, we found that the contents of both superoxide and H_2_O_2_ were maintained at lower levels upon ethylene treatment, while those in the control plants were remarkably promoted by ethylene. Furthermore, overexpression of *TERF1* increased tobacco tolerance to H_2_O_2_ during seedling development. These findings indicate that TERF1 may play a role in the regulation of ROS production or scavenging. Plant cells contain multiple enzymes for ROS scavenging, including superoxide dismutase (SOD), ascorbate peroxidase (APX), glutathione peroxidase (GPX), and catalase (CAT)^1^,^72^,^73^,^74^. SODs act as enzymes catalyzing the transformation of highly damaging superoxide to H_2_O_2_, and APX, GPX, and CAT function in H_2_O_2_ detoxifying^1^,^72^,^73^,^74^. Previous studies showed that ERF transcription factors could regulate the transcription of ROS metabolic enzymes to change plant ROS tolerance^39^,^48^,^49^,^50^,^51^. The transcriptional analyses in this study revealed that overexpression of *TERF1* enhanced the transcription of *NtCAT* and *NtGPX*, which function in plant ROS scavenging, and activated the expression of *NtCA* which encodes a carbonic anhydrase that could enhance plant hypersensitive defense to ROS stress^63^. Ectopic expression of *TERF1* also increased the transcription level of *NtVCT* that encodes GDP-D-mannose pyrophosphorylase, an enzyme catalyzing the biosynthesis of ascorbic acid. And, ascorbic acid, known as vitamin C, is an important substance in plant ROS tolerance^66^,^67^,^72^. Previously, a TERF1 homologous ERF transcription factor JERF3 was found to activate the expression of *NtCA* via binding the cis-element in *NtCA* promoter^39^. TERF1 might regulate the transcription of *NtCA* in similar manners. The transgenic plants in this study were previously developed lines with high-level ectopic *TERF1* expression, and their growth was a bit slower than control plants under normal conditions due to the activation of ethylene responses^52^. This phenomenon was also observed in this research, however, their growth rate and survival capability were apparently higher than control plants upon H_2_O_2_ treatment. These finding imply an involvement of ROS scavenging gene in the growth control. We ever determined if the internal transcription of tobacco *ERF1* gene was altered by *TERF1*-expressing, but found no significant change under normal condition or ethylene induction (data not shown). Cumulatively, these results imply that TERF1 may regulate diverse aspects in plant ROS responses through modulating the transcription of genes related to ROS metabolism.

This work revealed that TERF1 is involved in ROS responses, and expression of *TERF1* could decrease ROS accumulation in tobacco plants and increase tobacco tolerance to H_2_O_2_ stress. We presume that ethylene signal pathway may play important roles in the regulation of both ROS production and scavenging. That is the reason that some investigation proved the involvement of ethylene in ROS production amplification^24^,^27^, while a number of ERF transcription factors have been found functioning in ROS scavenging^39^,^48^,^49^,^51^. Moreover, ERF transcription factors could regulate plant responses to multiple stresses^38^,^39^,^48^,^49^, and their functions in ROS responses may be modulated by various signaling pathways. These facts suggest that ERF transcription factors coordinate a complicated regulatory network between ethylene signaling and ROS responses.

## Materials and Methods

### Plant material and growth conditions

Tomato (*Lycopersicon esculentum* cv Lichun) seedlings were grown in an indoor growth room at 25 °C under a 16 hr light/8 hr dark photoperiod as previously described^52^,^53^. For ROS induction of *TERF1* transcription, 4-week-old tomato seedlings were treated by spraying with 50 μm MV or 100 μm H_2_O_2_ in 0.1% Tween 20 solution as described by Wu *et al*.^39^, and then collected for total RNA preparation at indicated time points.

Tobacco (*Nicotiana tabacum* cv NC89) plants were grown in the same indoor growth room. The transgenic tobacco plants expressing *TERF1* were previously generated by Huang *et al*.^52^. T_3_ transgenic tobacco plants were used for the experiments. Leaves of 6-week-old tobacco seedlings collected for transcriptional assays. For H_2_O_2_ induced transcription assay, leaf samples were collected after 6 hr of treatment with H_2_O_2_ as described above.

### Yeast-one-hybrid assay

The reporter plasmids were constructed as described by Wang *et al*.^75^. The PERE-like-element-containing fragment in *TERF1* promoter was amplified with primer 5′-TCTTTAATTATAGATATTTTAAAC-3′ and 5′-CGTTAGTACTTATTTGAATGTATC-3′, and cloned as a trimer into the MCS upstream of *HIS3* minimal promoter in pHISi-1 vector and that upstream of the *lacZ* minimal promoter in pLacZi vector (Clontech, USA). Then, these two plasmids were linearized and co-transformed into yeast stain YM4271 to obtain the reporter yeast. The cDNAs of *LeEILs* were cloned with following primers: 5′-TCATCCTGTGGAAGATGATGATGT-3′ and 5′-ACAACATGTCAACAGACTTCTGGC-3′ for *LeEIL1*, 5′-TGGCTGCCAAGATGATGATGTTTG-3′ and 5′-CTTGATGTTCATTTGAGTAATCGC-3′ *LeEIL2*, 5′-TGGTAAATGGGGATATTTGAAGAT-3′ and 5′-CAGTTTAATACTAGTACTAGTTCA-3′ for *LeEIL3*, and 5′-GAGTTTGTTCTTGTGAAGATGATG-3′ and 5′-ACGTTTCACCAATATCATGGCTAG-3′ for *LeEIL4*. Then, the coding sequences of *LeEILs* were fused into downstream of transcriptional activation domain of the yeast vector pB42AD (Clontech, USA), and introduced into the yeast reporter strain respectively. Transformants that could grow on the selective medium lacking histidine but containing 30 mM 3-AT were subjected to filter-lifted β-galactosidase activity according to the manufacturer’s protocol (Clontech, USA).

### Gel-shift assay

The coding sequence of LeEIL4 was cloned into the vector pGEX-4T-1 (Amersham, USA), and transformed into *Escherichia coli* BL21 cells to express the fusion protein GST-LeEIL4. The recombinant protein was purified via GSH-affinity chromatography, and the GST protein was obtained in the same way to act as control. Gel-shift assay was performed using the DIG Gel Shift Kit (Roche, Germany) with the PERE-like elements in *TERF1* promoter as probes. Sequences of the probes are 5′-TATAGATATTTTAAACATTTTGAATTATCAATTATTGTGA-3′ for element at position -920–920, 5′-ATAAATATATAAATTTCATTCAAAAAAAATTGAAGATCTC-3′ for element at position -857–851, and 5′-ATAAATTGAACGATACATTCAAATAAGTACTAACGATTAT-3′ for element at position -787–781, in which the core sequences of PERE-like elements are underlined. Probe labeling with DIG-ddUTP, and the preparation and separation of binding reactions were according to the manufacture’s instruction. In the competitive binding assay reactions, a 200-fold excess of the corresponding unlabeled probes were added as specific competitors. After separation in polyacrylamide gel, the probes were transferred onto nylon membrane and crosslinked under UV light, and then detected using anti-Digoxigenin-conjugated alkaline phosphatase included in the kit.

### *Agrobacterium*-mediated transient transcription activation assay

To construct the reporter vector, cauliflower mosaic virus 35S promoter in pCAMBIA1381 was replaced with the *TERF1* promoter (-1420–1 bp) cloned previously by Li *et al*.^53^ to generate *TERF1*-promoter-controlled *GUS* expressing vector. To construct effector vectors, the coding sequences of *LeEILs* were introduced into pCAMBIA1381 to replace the *GUS* gene under the control of CaMV 35S promoter. The obtained plasmids were then introduced into *Agrobacterium tumefaciens* strain LBA4404. *Agrobacterium*-mediated transient transcription activation assay was performed with tobacco leaves from 6-week-old wild type tobacco seedlings as described previously by Wu *et al*.^39^. The GUS activity was measured 48 hr after infiltration as described by Wu *et al*.^39^.

### Tobacco seedling development under H_2_O_2_ stress

For seedling development assay under H_2_O_2_ stress, tobacco seeds were surface sterilized, sowed onto plates with MS (Murashige and Skoog) medium, and kept at 4 °C for 3 days to break the dormancy before moving to the indoor growth room. After germination in the growth room, the seeds were transferred onto MS medium plates supplemented with desired concentration of H_2_O_2_ and cultured in the same indoor growth room. The survival rate of tobacco seedlings was measured after 14 days of culture on MS medium supplemented with H_2_O_2_, and the average fresh weight of seedling was calculated by measuring ten seedlings of each plant set meanwhile.

### Detection of ROS accumulation in tobacco leaves

To analyze the ROS accumulation in tobacco leaves, 6-week-old wild type and *TERF1*-expressing tobacco plants were placed in an 18 L plastic chamber to be treated with ethylene gas (200 ppm), and tobacco leaves was harvested for ROS determination after 3 hr of treatment. Tobacco leaves were stained with nitroblue tetrazolium (NBT) and diaminobenzidine tetrahydrochloride (DAB) to detect the contents of superoxide and H_2_O_2_ respectively as described by Lee *et al*.^76^.

### Transcriptional analyses

Total RNAs were extracted using Trizol reagent (Invitrogen, USA). The first strand cDNA was synthesized with M-MLV reverse transcriptase (Promega, USA) and 1 μg of total RNAs according to the manufacturer’s instructions. Quantitative RT-PCR amplifications were carried out using the following gene-specific primers: 5′-ATGTCAAGCCCACTAGAGAT-3′ and 5′-CTATGATGAAGTCATTAAAAGC-3′ for *TERF1* (AY044236); 5′-GCATTGGATACGATACCACACCA-3′ and 5′-GCAGACGAGTACATGGGATCTTT-3′ for *LeEIL1* (AF328784); 5′-TGATGACGTGACGAAGCAAGATG-3′ and 5′-TGCACGATCCTCCAACCTCTACA-3′ for *LeEIL2* (AF328785); 5′-TCAACTTGGGAGGAAGCGACTAC-3′ and 5′GCACTTACAACCATGGAAACATC-3′ for *LeEIL3* (AF328786); 5′-CAGTCCACCAATTACCCTGGAAG-3′ and 5′-GACTGACTATATACGTTTCACCA-3′ for *LeEIL4* (AB108840); 5′-CATGCCATTCTCCGTCTTGA-3′ and 5′-GCTAGGAGCCAATGCAGT-3′ for tomato actin gene *Tom41* (U60480); 5′-CTGAGAAATATGAGAAGAACC-3′ and 5′-GAAAGACCGAACTCAAGTC-3′ for *NtCA* (AF454759); 5′-GGAGTCCGCATCAAGAAACA-3′ and 5′-CACATAACTATTTCAGGTTTCA-3′ for *NtVTC1* (AB066279); 5′-TGGATCTCATACTGGTCTCA-3′ and 5′-TTCCATTGTTTCAGTCATTCA-3′ for *NtCAT* (U93244); 5′-GGTTTGCACTCGCTTCAAG-3′ and 5′-AGTAGTGGCAAAACAGGAAG-3′ for *NtGPX* (AB041518); 5′-CCACACAGGTGTGATGGTTG-3′ and 5′-CACGTCGCACTTCATGATCG-3′ for *NtActin* (X63603). Actin genes were used as the internal control.

## Additional Information

**How to cite this article**: Zhang, H. *et al*. Ethylene Response Factor TERF1, Regulated by ETHYLENE-INSENSITIVE3-like Factors, Functions in Reactive Oxygen Species (ROS) Scavenging in Tobacco (*Nicotiana tabacum* L.). *Sci. Rep.* **6**, 29948; doi: 10.1038/srep29948 (2016).

## Supplementary Material

Supplementary Information
