# Supplementary material for: Ethylene Response Factor TERF1, Regulated by ETHYLENE-INSENSITIVE3-like Factors, Functions in Reactive Oxygen Species (ROS) Scavenging in Tobacco (Nicotiana tabacum L.)
Source: Sci Rep. 2016 Jul 20;6:29948. doi: 10.1038/srep29948 (PMC4951782; doi:10.1038/srep29948)
Supplement: Supplementary Information [file srep29948-s1.doc]

**Ethylene Response Factor TERF1, Regulated by** [**ETHYLENE-INSENSITIVE3-like Factors,**](http://www.ncbi.nlm.nih.gov/pubmed/21914654) **Functions in Reactive Oxygen Species (ROS) Scavenging in Tobacco (*Nicotiana tabacum* L.)**

Hongbo Zhang^1,#,*^, Ang Li^2,3,#^, Zhijin Zhang^2^, Zejun Huang^4^, Pingli Lu^5^, Dingyu Zhang^1^, Xinmin Liu^1^, Zhong-Feng Zhang^1^, Rongfeng Huang^2,*^

^1^Tobacco Research Institute, Chinese Academy of Agricultural Sciences, Qingdao 266101, China;

^2^Biotechnology Research Institute, Chinese Academy of Agricultural Sciences, Beijing 100081, China;

^3^Institute of Crop Sciences, Chinese Academy of Agricultural Sciences, Beijing 100081, China;

^4^The Institute of Vegetables and Flowers, Chinese Academy of Agricultural Sciences, Beijing 100081, China;

^5^Institute of Plant Biology, School of Life Sciences, Fudan University, Shanghai 200433, China.

^#^These authors contributed equally to this work.

^*^To whom correspondence should be addressed: E-mail: [zhanghongbo@caas.cn](mailto:zhanghongbo@caas.cn); [rfhuang@caas.cn](mailto:rfhuang@caas.cn).

**Supplementary Figure**


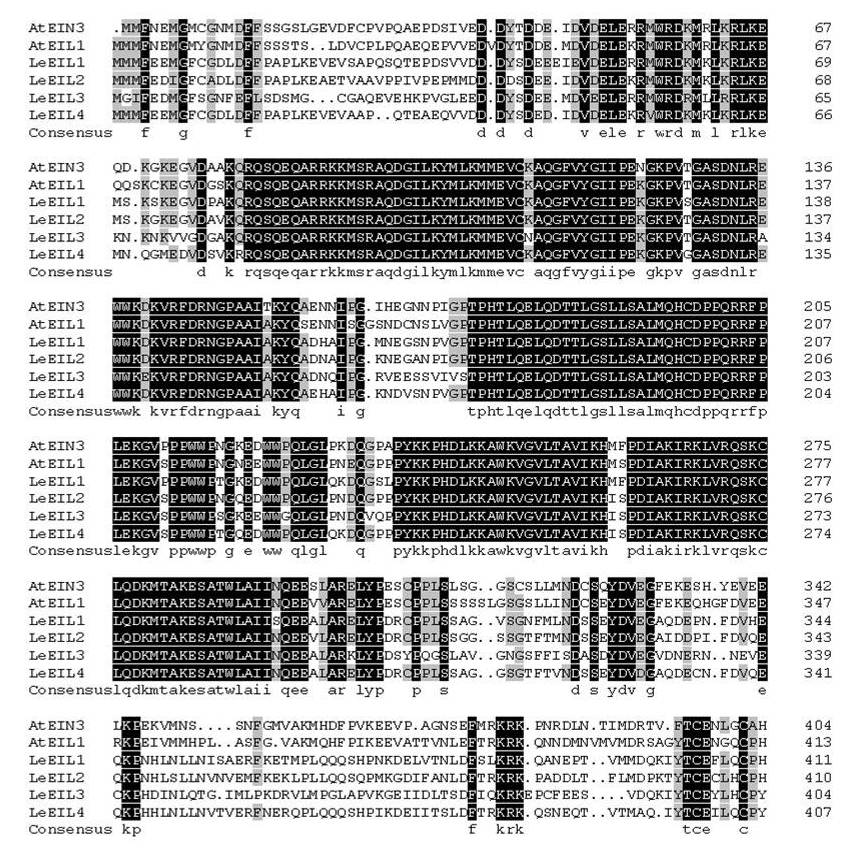


**Figure S1. Alignment of amino acid sequences of LeEILs and Arabidopsis EIN3/EIL1.** Shaded boxes indicate the degrees of amino acid sequence identity (dark gray, 100%; medium gray, 75%; light gray, 50%). Genbank accessions: AF004216 (AtEIN3), AF004213 (AtEIL1), AF328784 (LeEIL1), AF328785 (LeEIL2), AF328786 (LeEIL3), AB108840 (LeEIL4).
